# Epigenetics and Malaria Susceptibility/Protection: A Missing Piece of the Puzzle

**DOI:** 10.3389/fimmu.2018.01733

**Published:** 2018-08-03

**Authors:** Charles Arama, Jaclyn E. Quin, Bourèma Kouriba, Ann-Kristin Östlund Farrants, Marita Troye-Blomberg, Ogobara K. Doumbo

**Affiliations:** ^1^Malaria Research and Training Centre, Department of Epidemiology of Parasitic Diseases, International Center of Excellence in Research, University of Sciences, Technique and Technology of Bamako, Bamako, Mali; ^2^Department of Molecular Biosciences, The Wenner-Gren Institute, Stockholm University, Stockholm, Sweden

**Keywords:** epigenetic, immunity, malaria, falciparum, protection, susceptibility

## Abstract

A better understanding of stable changes in regulation of gene expression that result from epigenetic events is of great relevance in the development of strategies to prevent and treat infectious diseases. Histone modification and DNA methylation are key epigenetic mechanisms that can be regarded as marks, which ensure an accurate transmission of the chromatin states and gene expression profiles over generations of cells. There is an increasing list of these modifications, and the complexity of their action is just beginning to be understood. It is clear that the epigenetic landscape plays a fundamental role in most biological processes that involve the manipulation and expression of DNA. Although the molecular mechanism of gene regulation is relatively well understood, the hierarchical order of events and dependencies that lead to protection against infection remain largely unknown. In this review, we propose that host epigenetics is an essential, though relatively under studied, factor in the protection or susceptibility to malaria.

## Introduction

Complex infectious diseases such as malaria, in which environmental and clinical features along with genetic susceptibility factors contribute significantly to the pathology of the disease, pose a great challenge for the identification of relevant biomarkers for protection against the disease. In the human host, the disease outcome is determined by a complex relationship between the host, the parasite, and the environment (1). For example, parasite virulence, infection burden, the route of inoculation, host’s immunity and susceptibility to infection, nutrition and gut microbiota, previous exposure to antimalarial drugs (2), or coinfection, such as with helminthes, virus, and bacteria (3) may influence disease severity. The contribution of immunogenetic factors to resistance against malaria has been thoroughly investigated (4, 5). However, immunogenetic biomarkers that significantly correlate with a more protective immune response against malaria are not yet available. Recent studies generating large sequencing and immune variable data sets have begun to provide valuable insights (6). However, to date, a lack of data has hampered the identification of functional genomic features as well as the discovery of specific roles of genes in malaria.

Our understanding of the role of epigenetics in complex disease is rapidly emerging. Epigenetics play an important role in hematopoiesis, for example, proliferation of hematopoietic stem cells, as well as the successive stages of differentiation into more committed progenitors, are regulated at the transcriptional level through epigenetic modifications (7). Although many works in epigenetics have been conducted in the field of hematopoietic cancers and autoimmune diseases, there is still a gap of knowledge of how these epigenetics mechanisms contribute to the susceptibility or resistance to infectious diseases such as malaria.

In this review, we present and discuss the potential role of epigenetic factors in the protection/susceptibility to diseases. Particularly, we reviewed how the current knowledge on how epigenetic regulation contributes to protective immunity and susceptibility to disease can be applied to address the challenge of malaria.

## Genetic Variations and Susceptibility to Malaria

Knowledge of the host genetic susceptibility to malaria is key to understanding the complexity of the host immune response and its interaction with parasite infection. *Plasmodium* has been a major cause of morbidity and mortality throughout human history. As a result of this, malaria is believed to have exerted evolutionary pressure on the human genome by selecting genetic polymorphisms that provide protection against severe disease (8). Hence, many studies have attempted to assess host genetic factors involved in both the host immune response to malaria and the disease outcome. The best examples of such diseases are hemoglobinopathies, in which hemoglobin S was the first described human host genetic factor associated with protection against malaria (9). Studies have shown that HbAS has a 90% protective effect against severe and lethal malaria (10) and 50% protective effect against mild clinical cases (11). In addition, the carriage of HbAS was associated with a significant delay in the time to first malaria clinical episode (12). Other hemoglobinopathies, which may have important role in the protection against malaria, include homozygote and heterozygote α-thalassemia. These genetic modifications showed a decreased risk of severe malaria in a systematic review and meta-analysis study (13). Genetic variations such as polymorphism in hemoglobin, intracellular enzymes, red-blood cell (RBC) channels, RBC-surface markers, and proteins impacting the RBC cytoskeleton and RBC morphology have also been shown to attenuate malaria pathogenesis (14). The RBC surface protein Duffy antigen receptor for chemokines (DARC) gene is one of the most compelling pieces of evidence for RBC evolution against *Plasmodim vivax* malaria. Mutation of the *DARC* gene is common among individuals in West and Central Africa and confers protection against *P. vivax* (15). However, recent evidence shows that *P. vivax* infects DARC negative individuals (16, 17). Therefore, understanding the molecular basis of genetic variations arising from selective pressure by malaria in different ethnic groups may offer insight into protective mechanisms against malaria pathogenesis.

## Epigenome-Wide Association Studies (EWAS) for Common Human Diseases

Epigenetics study the mechanisms that determine and/or perpetuate genomic functions without changes in DNA sequence (18). It consists of the collective changes in phenotype due to processes that arise independently of primary DNA sequence (18).

During the past decades, genome-wide association studies have incrementally provided evidence of the association between genetic variations at a whole genome level and susceptibility to human diseases (19). However, genetic variation alone has not been able to give a clear explanation of the complex interaction between the genomic expression and the outcome of certain diseases. The impact of environmental factors on manifestation of disease may be the reason for these limitations. Particularly, different environmental conditions can result in the establishment of different epigenetic states responsible for mediating gene expression patterns and other genomic responses. This makes the epigenome an especially intriguing and interesting target to study. Recent technological advances in high-throughput genomic analysis have improved the genome-wide examination of epigenetic modifications such as DNA methylation and histone modification, collectively referred to as EWAS. These have enabled unprecedented systematic large-scale association testing in correlation with disease phenotypes. Importantly, EWAS have begun to establish the link between variation in epigenetic regulation and susceptibility to disease, including autoimmune diseases such as rheumatoid arthritis (20) and type I diabetes (21).

The difficulty in EWAS arises in the interpretation of the findings. For example, vastly different epigenetic patterns exist in distinct cell types, and thus, cell subtype effects account for a major proportion of the epigenetic changes associated with disease phenotypes (22). To date, EWAS represent an important contribution toward a better understanding of the etiological role of epigenetic variations in autoimmune diseases; however, more evidence is needed to establish the relationship with more complex infectious diseases. However, in malaria, researchers have only just begun to perform genome wide examination of epigenetic variations and protection from malaria in different ethnic groups (23). It is the ambition that further advances will help account for our gap in knowledge of what underlies the differences in clinical phenotypes of certain complex infectious diseases including malaria, tuberculosis, and AIDS.

## Epigenetics Mechanisms and Acquisition of Protective Immunity

Epigenetic changes underlie both the differentiation and activation of immune cells, which are regulated by precise spatial and temporal control of gene expression (7). For example, hematopoietic stem cell proliferation and differentiation into different immune cell types requires changes in chromatin structures and nuclear architecture, which depend on complex epigenetic regulation (24–26). The importance of epigenetic processes for the function of the immune system is illustrated by the prevalence of mutations in hematopoietic epigenetic regulators in leukemia and lymphoma (27–30), as well as identification of somatic mutations of epigenetic regulators in autoimmune diseases and other immune-based disorders (31–36). Environmental exposures throughout the life span induce genetic and epigenetic alterations, particularly in susceptible populations (37). This indicates that epigenetics may also hold the key to a larger understanding of the contributing factors of human health, where early life events shape later susceptibility to disease.

The major chromatin changes in immune cells occur by DNA methylation and histone modification, but also by rearranging chromatin structure. The primary DNA modification is 5-methylcytosine (5meC) DNA methylation, arising from transfer of a methyl moiety from *S*-adenosylmethionine to the 5-position of cytosine in certain CpG dinucleotides with the help of the DNA methyltransferases (DNMTs). Other epigenetic DNA modifications consist of the conversion of 5-methylcytosine to 5-hydroxymethylcytosine, and adenine methylation (38). The DNA methylation pattern changes often during hematopoiesis to silence some genes by introducing CpG methylations and activate others by removing DNA methylations. Posttranslational modifications of histones such as acetylation and methylation are important in regulating the transcriptional activity of cells. They occur in a site-specific manner that influences the binding and activities of other proteins and chromatin organization (39). “Writers” such as histone acetyltransferases and methyltransferases (HMTs) catalyze histone acetylation and methylation while “erasers” such as the histone deacetylases (HDACs) and histone demethylases result in the removal modifications within the chromatin. The epigenetic modification of chromatin is precisely regulated *via* mediating the activity and recruitment of these enzymes to specific loci, resulting in specific changes in gene expression, chromatin organization, and other DNA regulatory processes, for example, *via* establishing co-regulatory transcription programs or specialized functional domains within the nucleus. These are also regulated by chromatin remodeling complexes that alter the density of chromatin (40).

Recently, it has been shown that epigenetic modifications regulate the expression of key immune system genes, underlying both the innate and adaptive immune responses (41, 42). In the adaptive immune response, the changes in phenotype that accompany T- and B-cell activation and differentiation are mediated through acquired transcriptional regulatory mechanisms, including epigenetic modifications resulting in distinct DNA methylation and histone modification patterns (43–46). For example, genome-wide DNA methylation analysis of T- and B-cells reveals distinct differences during the transition from naive to effector cells (47, 48). In memory T-cells, histone modifications epigenetically mark genes and prime them for rapid and robust transcription following exposure to specific antigens (45). In different T-cell populations, specific regions are differentially methylated, for example, the *CD4* gene was hypermethylated in CD8^+^ T-cells and hypomethylated in CD4^+^ T-cells (48), while differential methylation of interferon gamma (IFN-γ) mediates differentiation of Th1 and Th2 cells (49). In contrast, in B-cell activation, DNA is predominantly hypomethylated (50). Epigenetic mechanisms also enable somatic hypermutation and class switch DNA recombination (51), thereby mediating antibody responses. Understanding the molecular mechanisms of these epigenetic changes in the memory responses of T- and B-cells may offer new areas in the development of safer and more effective vaccines (43).

In addition to classical adaptive immune memory, the innate immune system also has a memory, which manifests as a previous challenge driving an increased (“trained”) or decreased (“tolerized”) response to a second challenge in comparison to naïve cells. This altered state can persist for weeks to months following the initial stimuli and results in cells of the innate immune system, including monocytes and macrophages, being more or less capable of producing inflammatory cytokines, and/or phagocytizing and killing microorganisms, in response to a second unrelated stimuli. On one side, “tolerance” can arise following high bacterial burden, preventing responsiveness to an additional challenge, or the immunosuppressive phenotype observed in late sepsis and is viewed as a strategy to limit inflammation (52). At the other end of the spectrum, “trained” immunity can be induced following certain live vaccinations (BCG vaccination is the best characterized example), infectious stimuli, or metabolites, and is characterized by a change in cellular metabolism from oxidative phosphorylation toward aerobic glycolysis, an increased proinflammatory response, and resistance to infection (53, 54). The main mechanism by which innate cells develop a memory is through long-term epigenetic reprogramming (54, 55). Tolerance and trained immunity are associated with distinct and opposing epigenomic states (56). For example, monocytes tolerized by LPS treatments are associated with H3K4 monomethyaltion and a failure to accumulate H3K27 acetylation and active histone marks at the promoters of tolerized genes, such as in the lipid metabolism and phagocytic pathways, during a second challenge (57, 58). On the other hand, monocytes trained by exposure to β-glucan are associated with H3K4 trimethylation and H3K27 acetylation at the promoters of genes, enabling higher transcriptional levels in genes such as pathogen-recognition receptors, signaling molecules, and proinflammatory cytokines (57, 59).

## Epigenetics and Malaria

**Figure 1:**
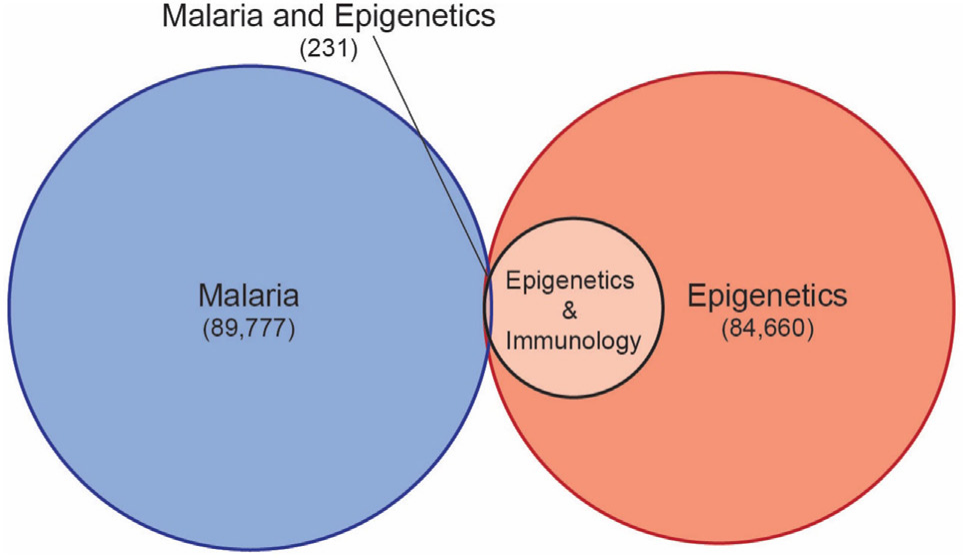
A database search of the Web of Science Core Collection for all publications with the topic of malaria (search term malaria*), epigenetics (search term epigen*), and epigenetics and immunology (search term epigen* and immun*), identified 12,484 publications addressing epigenetics and immunology, but only 231 publications addressing epigenetics and malaria (search term malaria* and epigen*) (https://www.webofknowledge.com accessed 20 June 2018).

Despite understanding that host epigenetics underlie differentiation and activation of immune cells, as well as the regulation of key genes in both the innate and adaptive immune responses, there is a vast gap of knowledge of role of epigenetic factors in the protection from or susceptibility to malaria. A systematic database search of all relevant publications illustrates how under studied the role of host epigenetics in malaria has been until very recently (Figure 1). Only 231 publications were identified that address both malaria and epigenetics, and of these, the vast majority address the role of epigenetics in gene regulation in the malaria parasite. Current knowledge of the role of epigenetics in *Plasmodium* biology, and how this may be exploited to combat malaria, has been comprehensively covered by recent reviews, and will not be discussed in depth here (60, 61).

The few research articles that address epigenetics of the infected host are not comprehensive but apply current knowledge of epigenetics and infection to specific questions in malaria. They investigated the epigenetic regulation of specific host genes that provide resistance to malaria (62) or mediate the immune response to malaria (e.g., promoter DNA methylation of TLR6 (63) and ABCB1 (64), and H3K4me3 at TNF, IL-6, and mTOR promoters (65)). Recently, Lessard et al. utilized epigenomic profiling to determine functionally relevant genomic sequences at a loci associated with protection from malaria (66). Others discussed the role of epigenetics in generating specific immune cell populations that contribute to susceptibility to or protection from malaria, particularly, CD4^+^ T-cells (67), and CD8^+^ T-cells (68), or the mechanisms by which testosterone (69) or nutrition (70) can confer susceptibility to or protection from malaria. Kumari et al. investigated the effect of the antimalarial drug artemisinin on epigenetic modifiers (71). Others examined global changes in miRNA expression (72) or DNA methylation in specific cell or tissue types of malaria-infected individuals (23, 64, 73). The majority of these research articles have been published within the last 2 years, hopefully indicating an interest in applying the lessons we have learned regarding the important role of epigenetics in other diseases to the challenge of malaria.

## Epigenetics and Protection from Malaria

Despite the few studies that directly address the role of host epigenetics in protection from or susceptibility to malaria, a number of recent studies indicate that immune responses that are controlled by epigenetic changes are important for protection from the disease. There is growing evidence that *Plasmodium* can induce a state of trained innate immunity. Stimulation of human peripheral blood mononuclear cells (PBMCs) with *Plasmodium falciparum*, both *in vivo* and *in vitro*, results in subsequent toll-like receptor specific stimuli driving significantly higher proinflammatory responses (74, 75). The hyper-responsiveness of PBMCs that have been exposed to either *P. falciparum*-infected RBCs or hemozoin is associated with increased H3K4 trimethylation at specific immuno-metabolic promoters (65). This trained immunity may be associated with protection from malaria. In *P. falciparum* malaria, patients who display the production of malaria-specific IFN-γ by PBMCs have significantly lower rates of reinfection (76, 77). Further, a recent study of the Fulani, an ethnic group with lower susceptibility to malaria, found that following *P. falciparum* infection, the Fulani displayed characteristics suggestive of trained immunity, with more transcriptionally reactive monocytes and a more pro-inflammatory response relative to a sympatric ethnic group (72). Finally, in mice BCG vaccination, which is known to induce trained innate immunity, results in reduced parasitemia during a subsequent challenge with malaria parasites (78). Conversely, malaria may induce innate immune tolerance, particularly, after multiple infections or in patients in which the infection progresses without treatment (79). Individuals historically infected with malaria as therapy for neurosyphilis exhibited depressed responses to a subsequent challenge with heat-killed *Salmonella* (80). The parasite burden that initiates symptomatic malaria increases with multiple infections, with individuals in endemic areas infected with *P. falciparum* for weeks to months while remaining apparently healthy (81, 82). Therefore, the dynamics of epigenetic regulation of innate immune memory, to either a “trained” or “tolerized” phenotype, has implications for the outcome of the disease.

A role for epigenetics in protection from malaria may also extend to nutrition, microbiome, and other factors that affect the metabolome. The role of dietary nutrients and gut microbiota in influencing immune function has now been well established (83–85). For example, probiotic commensal bacteria can dampen immune activation, protecting against allergy development (86, 87). The different activation of immune cells is underscored by epigenetic changes (88–90). In the context of malaria, gut microbiota has a potential role in resistance of individuals to malaria (91). Particularly, the role of dietary advanced glycation end-products in the modulation of immune responses through chronic oxidative stress, which mediates epigenetic and transcriptional programs, has been established, which also seems to play a role in the natural protection against malaria (92).

Whole blood genomic DNA studies of global DNA methylation in individuals infected with *P. falciparum* have shown that global DNA methylation levels are inversely proportional to parasitemia, with reduced 5meC levels in infected compared to uninfected individuals (64). However, currently, it is unclear whether this is due to a strategy of the host immune system to mediate the immune response, or alternatively whether the parasite and/or its by-products can change methylation levels in the host genome to provide itself with a survival advantage.

## Epigenetics and Susceptibility to Malaria: The other Side of the Coin

Immunity to malaria is short-lived, despite repeated parasite exposure in endemic areas, and the established strong selective force of malaria on human populations. Malaria parasites have evolved to escape the immune system of the human host, utilizing a number of mechanisms including allelic variation and modification of host cell phenotype (93). Recently, the ability of the parasite to impact host immune response through modulating epigenetic and transcriptional pathways has been proposed. For example, malaria patients display reduced numbers of circulating dendritic cells (DCs) and an accumulation of immature DCs (94), and further studies have implicated the malaria pigment hemozoin in partially preventing DC maturation and capacity to activate T-cells (95–98). However, so far, the role of epigenetic mechanisms have not been established, only implied. For example, the malaria parasite and its by-products drive DNA hypomethylation and increased expression of the ABCB1 gene (99), and the hypothesis that this multidrug resistance transporter protein is regulated by malaria infection to eliminate hemoglobin degradation products is being investigated (64). In support of this, other parasites have demonstrated ability to co-opt host epigenetic mechanisms to orchestrate changes in host gene expression (100). For example, *Mycobacterium tuberculosis* inhibits IFN-γ-induced expression of several immune genes through histone acetylation, contributing to the persistence of long-term chronic tuberculosis infections in some patients (101–103). Other protozoan parasites specifically, including *Leishmania* and *Toxoplasm*a, employ a variety of strategies to actively modulate host immune epigenome and transcriptome. For example, *Leishmania* parasite replicates in the macrophages of its mammalian host, where it efficiently inhibits activation of innate immune reponses, such as antigen presentation, IFN-γ, and activation of cytokines and chemokines. Infection of macrophages with *Leishmania donovani*, compared to a heat-killed control, results in global changes in DNA methylation, including at genes involved in macrophage activation (104, 105). The intracellular parasite *Toxoplasma gondii* can prevent chromatin remodeling and association of transcription factors at the TNF-α promoter, and mediate levels of DNA methylation at the arginine vasopressin (*Avp*) gene promoter (103, 106, 107).

The other side of the coin is also related to the role of immune response in the pathology of malaria as a disease. Severe pathophysiological events during malaria infection include erythrocyte destruction and ineffective erythropoiesis, adhesion of *Plasmodium-*infected red blood cells to capillary veins of vital host organs, and excessive production and the release of proinflammatory cytokines (108). These symptoms are driven by high levels of proinflammatory immune responses. In escaping the host immunity, the parasite may also prevent the development of symptoms of severe malaria infection. Thus, understanding the epigenetic mechanisms by which the parasite stimulates or evades immune response in the human host may shed light into how this complex host–parasite interaction results in the pathology of the disease.

## Conclusion

Despite long lasting efforts to control and eliminate malaria infection, the disease remains a public health concern in sub-Saharan Africa. According the World Health Organization (WHO), malaria remains a major cause of morbidity and mortality, causing an estimated 445,000 deaths globally in 2016 (109). Sub-Saharan Africa is the most affected region with 92% of global malaria death. Among these, 88% occurs in children under 5 years of age (109). Challenges in malaria include difficulties in estimating the changing burden of disease due to limitations in health reporting systems in many African countries, inequality in malaria intervention coverage in countries with slow growth or a large baseline inequality (110), emergence of mosquitos resistant to insecticides, and the spread of artemisinin-resistant malaria parasites in Southeast Asia (111). Consequently, progress against malaria has stalled, with increased worldwide incidence of malaria reported for the first time in recent history (109).

The development of new strategies that will explore new research avenues and hypotheses on biological factors of malaria susceptibility or resistance are urgently needed. This should be a priority if the goal of eradication is to be achieved by 2030.

Interestingly, growing evidence suggests that epigenetics play a key role to multiple levels of this complex disease, including immune evasion by the parasite, tolerance, training, and adaptive responses. The tools and frameworks are readily available to investigate the impact of epigenetics in the protection from or susceptibility to malaria in more depth. In other fields, drugs targeting epigenetic enzymes and processes have already advanced to clinic. For example, the DNMT inhibitors and HDAC inhibitors have proven efficacious in the treatment of cancer, particularly hematopoietic cancers (29, 112, 113). Inhibitors of chromatin modifying enzymes that target the *Plasmodium* parasite have already been considered as an antimalarial strategy (61). Understanding how epigenetic mechanisms in the human host impact the disease outcome during malaria, through either driving or dampening immune responses, may offer a relatively achievable approach to develop new strategies that can be applied to the treatment of the disease.

There are several challenges that epigenetic epidemiological studies have to address to elucidate fully the role of epigenetics in susceptibility or protection to malaria. One of these challenges is to understand how the parasite alters the host immune responses by exerting a strong selective pressure on population genetics in endemic regions. A better understanding of the mechanisms that underlie the chromatin and DNA methylation changes are topics of scientific interest. Such studies will generate more evidence in the role of epigenetics in the acquisition of protective immunity against infectious disease such as malaria. As we gain insight into the functional significance of changes in DNA methylation events and other epigenetic mechanisms, there will be a push to manipulate these processes, as tools and strategies to develop vaccines and target for therapeutic discoveries.

## Author Contributions

CA: conceptualization, writing—original draft, writing—review and editing; BK and JQ: writing—review and editing; MT-B, A-KF, and OD: conceptualization, writing—review and editing. All authors read and approved the final manuscript and agreed to be accountable for all aspects of the work.

## Conflict of Interest Statement

The authors declare that the research was conducted in the absence of any commercial or financial relationships that could be construed as a potential conflict of interest.
